# The association of cigarette smoking and alcohol drinking with body mass index: a cross-sectional, population-based study among Chinese adult male twins

**DOI:** 10.1186/s12889-016-2967-3

**Published:** 2016-04-11

**Authors:** Chunxiao Liao, Wenjing Gao, Weihua Cao, Jun Lv, Canqing Yu, Shengfeng Wang, Bin Zhou, Zengchang Pang, Liming Cong, Zhong Dong, Fan Wu, Hua Wang, Xianping Wu, Guohong Jiang, Xiaojie Wang, Binyou Wang, Liming Li

**Affiliations:** Department of Epidemiology and Biostatistics, School of Public Health, Peking University, Beijing, 100191 China; Qingdao Center for Diseases Control and Prevention, Qingdao, 266033 China; Zhejiang Center for Disease Control and Prevention, Hangzhou, 310051 China; Beijing Center for Disease Control and Prevention, Beijing, 100013 China; Shanghai Center for Disease Control and Prevention, Shanghai, 200336 China; Jiangsu Center for Disease Control and Prevention, Nanjing, 210009 China; Sichuan Center for Disease Control and Prevention, Chengdu, 610041 China; Tianjin Center for Disease Control and Prevention, Tianjin, 300011 China; Qinghai Center for Disease Control and Prevention, Xining, 810007 China; Harbin Medical University, Harbin, 150081 China

**Keywords:** Twin studies, Body mass index, Gene-environment interaction, Smoking, Alcohol drinking

## Abstract

**Background:**

Obesity is a multifactorial abnormality which has an underlying genetic control but requires environmental influences to trigger. Numerous epidemiological studies have examined the roles of physical inactivity and dietary factors in obesity development. Interactions between obesity-related genes and these lifestyles have also been confirmed. However, less attention has been paid to these complex relationship between cigarette smoking, alcohol drinking and obesity. The purpose of this study was to assess whether cigarette smoking and alcohol drinking were associated with body mass index (BMI), and whether these lifestyle factors modified the genetic variance of BMI.

**Methods:**

Subjects were twins recruited through the Chinese National Twin Registry, aged 18 to 79 years, and the sample comprised 6121 complete male twin pairs. Information on height, weight, cigarette smoking and alcohol drinking status were assessed with self-report questionnaires. The associations of cigarette smoking and alcohol drinking with BMI were evaluated by linear regression models. Further, structure equation models were conducted to estimate whether cigarette smoking and alcohol drinking status modified the degree of genetic variance of BMI.

**Results:**

After adjustment for a variety of socio-demographic and lifestyle factors, former smokers had higher BMI (β = 0.475; 95 % CI, 0.196 to 0.754) whereas moderate to heavy smokers had lower BMI (β = −0.115; 95 % CI, −0.223 to −0.007) when compared with nonsmokers. BMI decreased with increased cigarette pack-years (β = −0.008; 95 % CI, −0.013 to −0.003). These effects still existed substantially in within-MZ twin pair analyses. By contrast, current alcohol drinking had no significant influence on BMI when additionally controlled for shared factors in within-pair analyses. Genetic modification by alcohol drinking was statistically significant for BMI (β = −0.137; 95 % CI, −0.215 to −0.058), with the intake of alcohol decreasing the additive genetic component of BMI.

**Conclusions:**

Cigarette smoking was negatively associated with BMI independent of genetic influences. The influence of genes on BMI was moderated by alcohol drinking, such that for individuals who were regular drinkers, genetic factors became less influential. Our findings highlight gene-alcohol interaction in finding candidate genes of BMI and elucidating the etiological factors of obesity.

## Background

Obesity has become a major global public health issue with great economic burden [1, 2]. Body mass index (BMI) has been largely used as a simple and convenient measure of obesity. Previous twin studies [3, 4] and recent genome-wide association (GWA) studies [5] have shown that BMI is highly influenced by genetic factors. In addition, lifestyle factors also play an extremely important role in determining BMI. Recent studies have shown that cigarette smoking and alcohol drinking can influence BMI and consequently affect the risk of being obesity. However, their relationships have remained inconclusive. Findings in some cross-sectional studies indicated that mean BMI of smokers tended to be lower than that of nonsmokers [6–8]. Nevertheless, other studies reported that smokers weighed more and had a higher risk to get obesity problem than nonsmokers [9, 10]. In terms of alcohol drinking, findings from large cross-sectional studies as well as from cohort studies with long periods of follow-up were not consistent, even findings from short-term experimental trials also did not show a clear parallel trend [11].

The complexity of the relationships among these genetic and environmental factors are generated by their interaction with each other rather than the respective factors act independently. The recent epidemic of obesity along with the increasing spread of unhealthy lifestyles worldwide [12, 13] is a good illustration of the concept of gene-environment interaction. Gene-environment interaction in obesity has been highlighted from observational studies and randomized intervention trials, which were mainly conducted in western countries [14]. Edwards et al. [15] found statistical significance in the interactions between variants in the adiponectin receptor 1 gene (ADIPOR1) and smoking among African Americans. In African-American smokers, the effect of ADIPOR1 was greatly reduced when compared with nonsmokers. With respect to alcohol drinking, it was reported that genetic risk influenced the association between alcohol consumption and central abdominal fat in British females [16]. However, due to the heterogeneity of the genetic background in obesity, whether cigarette smoking and alcohol consumption modify the genetic factors of obesity in Chinese population is largely unknown.

Twin design is seen as a useful method of controlling confounders in observational epidemiologic studies. Monozygotic (MZ) twins are perfectly matched for genetic and childhood family environmental factors while dizygotic (DZ) twins share on average half of their genes and all their family environment. Therefore, comparing twins within pairs can provide powerful control for genetic and shared environmental confounding factors, which are typically different among unrelated individuals [17].

Using structural equation modeling methods, twin studies can further evaluate how genetic variance changes as a function of environmental exposure [18]. For example, most studies have demonstrated reduced genetic effects on the variation of BMI in physically active subjects when compared with inactive subjects [19–21]. However, few studies have reported the genetic modification of cigarette smoking and alcohol drinking on BMI.

Since the impact of cigarette smoking and alcohol drinking and the interplay between these behavioral factors and genes in obesity are still not well understood especially in Chinese population, we aimed to examine the associations of cigarette smoking and alcohol drinking with obesity, as indexed by BMI in 18 to 79 years old male twins based on a large number of twin pairs from the Chinese National Twin Registry (CNTR). Further, we extended current study by examining whether cigarette smoking and alcohol drinking modified the genetic influences of BMI using gene-environment interaction models.

## Methods

### Study sample

The participants belong to the Chinese National Twin Registry (CNTR), the first and largest population-based twin registry in China described in detail elsewhere [22].

During 2011 to 2012, twin members were recruited in this study through an in-person interview by means of a questionnaire in 9 provinces or cities, including Jiangsu, Zhejiang, Sichuan, Heilongjiang and Qinghai province, Tianjin, Beijing, Qingdao and Shanghai City. For the purpose of this study, cigarette smoking and alcohol drinking data from male twins were used because the corresponding prevalence values were negligible in female twins. Twin pairs were eligible for the present study if (1) male twins aged between 18 and 79 years old, (2) weight, height, age and zygosity information from both twins of a pair were available, (3) free of cardiovascular heart disease, stroke, type 2 diabetes and cancer. Among these eligible twin pairs, 129 twin pairs reared apart were excluded from analyses (reared apart was defined according to SATSA’s definition as twins who had been reared apart for at least 1 year before the age of 11).

At last, the study population included 6121 complete male twin pairs (4122MZ and 1999DZ twin pairs). The determination of zygosity was based on age, gender, questions of appearance confused by strangers and previously perceived zygosity from questionnaires. This method has been validated using DNA genotyping from 192 pairs of same-gender twins and found to have an AUC of 89.03 % [23].

All participants provided informed consent and Biomedical Ethics Committee at Peking University, Beijing, China approved the study protocol.

### Measures

#### Explanatory variables

Cigarette smoking was coded into four categories (nonsmoker, former smoker, current light smoker, and current moderate to heavy smoker). Nonsmokers were defined as those who gave negative answers to ‘Do you smoke?’. Those who responded ‘I have quit smoking for one month or more’ were defined as former smokers. Current smokers were those who gave affirmative responses to ‘Do you smoke?’. The definition of current light and moderate to heavy smokers were current smokers who smoked one to 9 and 10 or more cigarettes daily, respectively. A continuous measure of cigarette smoking (cigarette pack-years) was also calculated (one “pack-year” is 20 cigarettes smoked/day for one year [24]) for current smokers. Alcohol drinking status was similarly defined depending on their responses to ‘Do you drink alcohol’. Those who gave affirmative responses were defined as current drinkers; former drinkers and nondrinkers were those who previously drank, but subsequently quit drinking for one month or more and who never drank before.

#### Outcome variable

Self-reported height and weight were used to calculate BMI. Data on height and weight were required to be accurate to the nearest centimeter and kilogram, respectively. BMI was defined as weight in kilograms divided by height in meters squared. The reliability of self-reported height and weight was assessed in a subsample of these twins who participated in a follow-up study in 2014 July whose body weight and height were measured by health-care professionals. Intraclass correlation for measured versus self-reported weight and height were .89 and .94, respectively, which suggested good reliability of self-reported BMI.

### Assessment of covariates

Potential covariates included age (18–79 y), region (‘south’: Jiangsu Province, Zhejiang Province, Shanghai City and Qingdao City, ‘north’: Heilongjiang Province, Tianjin City and Beijing City, ‘west’: Sichuan Province and Qinghai province), zygosity (MZ, DZ), education attainment (illiterate or primary education, secondary education and tertiary education), marital status (married, live alone) and regular physical activity at least 20 min in 5 days of a week (yes, no, unclear).

### Statistical methods

We compared epidemiological characteristics between MZ and DZ twins. *P*-values were corrected for the correlation between co-twins using multinomial logistic regression for categorical variables and mixed-effects models for continuous variables. Regression models and gene-environment interaction models were used to examine the associations of cigarette smoking and alcohol drinking status with BMI.

### Linear regression models

Mixed-effect linear regression models with a random intercept for each twin pair to account for twin clustering [25] were performed to estimate associations between cigarette smoking, alcohol drinking and BMI in the whole population treating twins as individuals. Covariates included age, region, zygosity, alcohol drinking, cigarette smoking, marital status, educational attainment and regular physical activity. Further, we applied fixed effect models separately for MZ and DZ twins to estimate the within-pair effects of cigarette smoking and alcohol drinking on BMI treating twins as pairs. Examining twins overall gives an average relationship between exposure and outcome across the twin population. If the association further persists in within-pair comparisons we can infer that something unique to each individual twin is contributing, rather than common to both twins. On the contrary, attenuation of the association in MZ or DZ twin pairs indicated that it was confounded by shared familial factors. Analyses were performed using Stata11.2 (Stata Corp, College Station, TX). *P*-values were two-sided and statistical significance was assumed at *P* < 0.05.

### Gene-environment interaction models

We used a univariate structural equation model to estimate the genetic and environmental influences on BMI variance. It is assumed that the variance of a total phenotype can be decomposed into three different sources of influence: additive genetic component (A), common environmental component (C) and unique environmental component (E). MZ twins share 100 % of their genes (at the sequence level), whereas on average, DZ twins share 50 % of their segregating genes. The correlation coefficient of A and C is 1.0 and 1.0 for MZ, and 0.5 and 1.0 for DZ, respectively. The proportion of variance explained by additive genetic factors is also commonly termed narrow-sense heritability. We first estimated the variance of BMI explained by A, C and E components. Nested model for which C was equated to zero was also fitted and Akaike Information Criterion (AIC) was used for comparison of goodness of fit of the models [26].

**Fig. 1:**
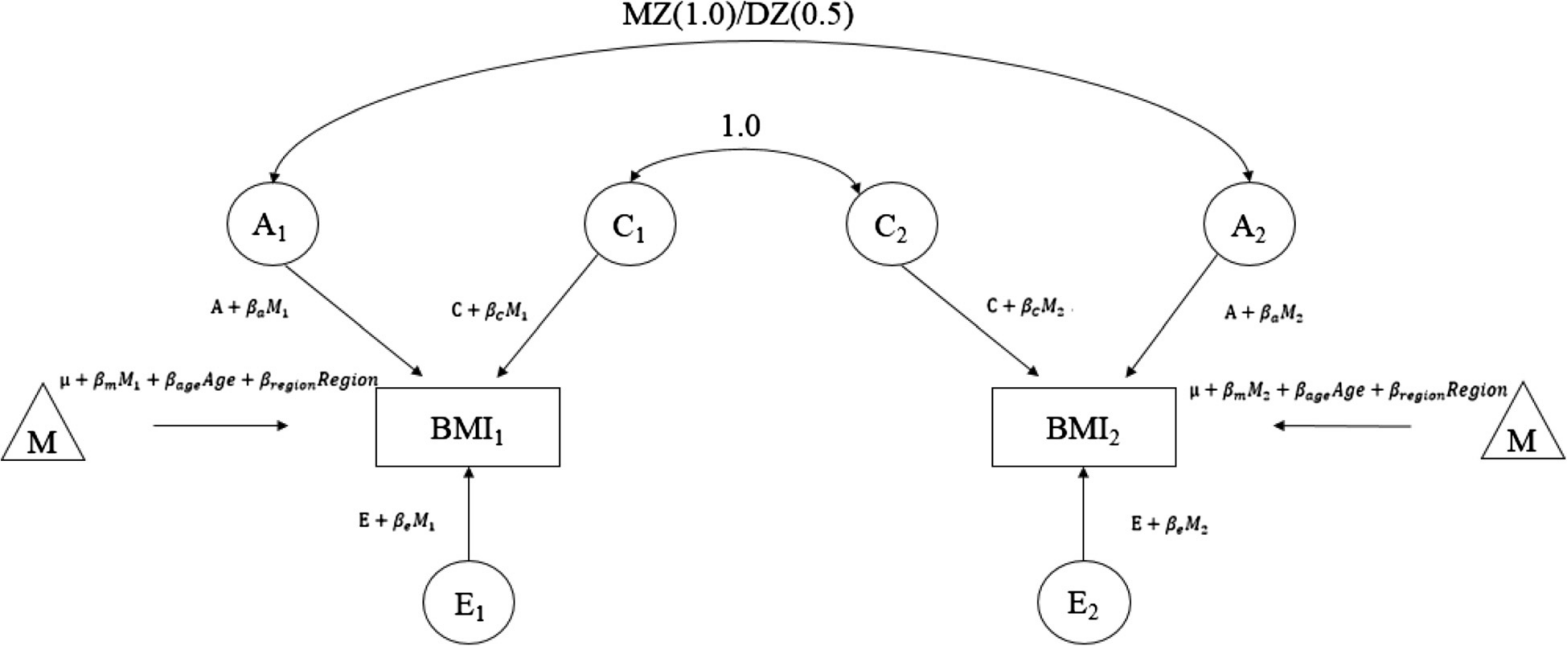
Gene-environment interaction model. M: environment moderator; BMI_1_ and BMI_2_: BMI in twin 1 and 2 within a twin pair, respectively; A_1_ and A_2_: additive genetic effects for twins 1 and 2 within a twin pair, respectively; C_1_ and C_2_: common environmental effects for twins 1 and 2 within a twin pair, respectively; E_1_ and E_2_: unique environmental effects for twins 1 and 2 within a twin pair, respectively; β_m_: mean moderator effect; β_a_: additive genetic moderator effect; β_c_: common environment moderator effect; β_e_: unique environment moderator effect

By assessing whether the genetic variance of BMI depends on certain lifestyles, we are able to offer in-depth insights into the possible gene-lifestyle interaction on BMI. Based on the best-fit model we conducted a gene-environment interaction model [18] (Fig. 1) to find whether genetic variance of BMI depended on certain lifestyles using moderate to heavy smoking (nonsmoking as reference) and current alcohol drinking (nondrinking as reference) as moderation factors (denoted as M). These factors can affect the BMI (β_m_) directly but can also modify the underlying genetic factors (β_a_), common environmental factors (β_c_) and unique environmental factors (β_e_) of BMI. The effects of moderation factors on genetic and/or environmental variance of BMI were evident when interaction parameters (β_a_, β_c_, β_e_) were significantly different from zero. We used a z-score to standardize BMI to have mean as 0 and variance as 1.

All model fitting analyses and maximum-likelihood parameter estimates were performed in OpenMx (Version 1.4), and all variance components were estimated with inclusion of age and region as covariates in the models.

## Results

### Descriptive characteristics of the study population

A total of 6121complete male twin pairs were included in this study of which 67.3 % were MZ twin pairs. The distribution of various epidemiological characteristics are presented in Table 1. Mean BMI of this population was 23.4 kg/m^2^ with a prevalence of obesity at 6.4 %. The proportion of current smokers were 44.3 % among which the average cigarette exposure was 15.2 pack years. 36.8 % of this population were moderate to heavy smokers (≥ 10 cigarettes a day). With respect to alcohol drinking status, 32.9 % of this population drank regularly. All these characteristics did not differ by zygosity (Table 1).

**Table 1:** Descriptive characteristics of the study population (*N* = 12242)^a^

	Total twins (*N* = 12242)	MZ twins (*N* = 8244)	DZ twins (*N* = 3998)	
	*N* (%)	*N* (%)	*N* (%)	*P* value^*^
Age, years (mean, SD)	38.8 (11.5)	38.9 (11.6)	38.7 (11.2)	0.550
BMI, kg/m^2 ^(mean, SD)	23.4 (2.9)	23.3 (2.9)	23.5 (2.9)	0.075
Overweight	3760 (30.7)	2529 (30.7)	1231 (30.8)	0.197
Obesity	785 (6.4)	507 (6.1)	278 (7.0)	0.066
Region				
South	8250 (67.4)	5622 (68.2)	2628 (65.7)	-
North	3240 (26.5)	2124 (25.8)	1116 (27.9)	0.060
West	752 (6.1)	498 (6.0)	254 (6.4)	0.445
Marital status				
Live alone	2839 (23.2)	1892 (23.0)	947 (23.7)	-
Married	9380 (76.6)	6334 (76.8)	3046 (76.2)	0.489
Educational attainment				
Primary	1681 (13.7)	1494 (18.1)	780 (19.5)	-
Secondary	8266 (67.5)	5637 (68.4)	2629 (65.8)	0.082
Tertiary	2274 (18.6)	1103 (13.4)	578 (14.5)	0.967
Cigarette smoking status				
Never	6561 (53.6)	4359 (53.4)	2162 (54.1)	-
Former	263 (2.1)	168 (2.0)	95 (2.4)	0.454
Current	5418 (44.3)	3677 (44.6)	1741 (43.5)	0.351
Light	917 (7.5)	588 (7.1)	329 (8.2)	0.072
Moderate to heavy	4501 (36.8)	3089 (37.5)	1412 (35.3)	0.064
Cigarette pack-years^b^ (mean, SD)	15.2 (12.7)	15.4 (12.9)	14.9 (12.4)	0.351
Alcohol drinking status				
Never	8035 (65.6)	5408 (65.6)	2627 (65.7)	-
Former	156 (1.3)	106 (1.3)	50 (1.3)	0.886
Current	4029 (32.9)	2717 (33.0)	1312 (32.8)	0.912
Regular physical activity				
No	5045 (41.2)	3392 (41.1)	1653 (41.3)	-
Yes	5661 (46.2)	3817 (46.3)	1844 (46.1)	0.877
Unclear	1511 (12.3)	1016 (12.3)	495 (12.4)	0.981

*Abbreviations: MZ* monozygotic, *DZ* dizygotic, *BMI* body mass index, *SD* standard deviation

^*^ *P* values were corrected for the correlation between co-twins using multinomial logistic regression for categorical variables and mixed-effects models for continuous variables

^a^Numbers may not sum to column totals due to missing data. Percentages may not add to 100 due to rounding

^b^Among current smokers only

### Associations between cigarette smoking, alcohol drinking and BMI

The results from linear regression analyses for the associations of cigarette smoking and alcohol drinking with BMI fitted in whole sample of twins as well as within MZ and DZ twin pairs are presented in Table 2. After adjusted for multiple factors, cigarette smoking as a continuous measure (cigarette pack-years) was negatively associated with BMI (β = −0.008; 95 % CI, −0.013 to −0.003), and the effect still existed substantially within MZ and DZ twin pairs. When compared with nonsmokers, former smokers had significantly higher BMI (β = 0.475; 95 % CI, 0.196 to 0.754) whereas moderate to heavy smokers had significantly lower BMI (β = −0.115; 95 % CI, −0.223 to −0.007). There was no attenuation of these associations and both coefficients were significant at .05 level in within-pair analyses of MZ twins.

**Table 2:** Adjusted effects (with 95 % CI and coefficient[β]) of cigarette smoking and alcohol drinking on BMI (*N* = 12242)

Lifestyle factors	All twins (Model 1)		Within MZ twin pairs (Model 2)		Within DZ twin pairs (Model 3)	
	β (95 % CI)	*P*	β (95 % CI)	*P*	β (95 % CI)	*P*
Cigarette pack-years^a^	−0.008 (−0.013, −0.003)	0.001	−0.007 (−0.014, −.0004)	0.038	−0.019 (−0.032, −0.005)	0.007
Cigarette smoking status (reference group: nonsmoker)^a^						
Former smoker	0.475 (0.196, 0.754)	0.001	0.376 (0.049, 0.704)	0.024	0.612 (−0.077, 1.300)	0.082
Light smoker	−0.008 (−0.178, 0.161)	0.926	0.122 (−0.092, 0.336)	0.264	−0.194 (−0.620, 0.233)	0.373
Moderate to heavy smoker	−0.115 (−0.223, −0.007)	0.037	−0.163 (−0.310, −0.017)	0.029	−0.357 (−0.642, −0.071)	0.014
Alcohol drinking status (reference group: nondrinker)^b^						
Former drinker	0.075 (−0.286, 0.435)	0.686	0.402 (0.003, 0.801)	0.048	−0.960 (−1.986, 0.067)	0.067
Current drinker	0.317 (0.203, 0.430)	< 0.001	0.096 (−0.066, 0.258)	0.245	0.307 (−0.008, 0.622)	0.056

*Abbreviations: MZ* monozygotic, *DZ* dizygotic, *BMI* body mass index, *CI* confidence interval

^a^Adjusted for age, region, zygosity, marital status, education level, alcohol drinking status and regular physical activity in three models

^b^Adjusted for age, region, zygosity, marital status, education level, cigarette smoking status and regular physical activity in three models

A positive relationship was observed between current alcohol drinking and BMI (β = 0.317; 95 % CI, 0.203 to 0.430) in the whole sample of twins. However, the effect was not significant in the within-pair analyses of MZ and DZ twin pairs indicating that the alcohol drinking-BMI association was due to shared familial factors. On the contrary, no significant difference in BMI was found when compared former alcohol drinkers with nondrinkers in the whole twin sample analysis. However, this difference became significant in within-MZ pair analysis.

### Moderating effects

We examined the modification effects of moderate to heavy cigarette smoking and current alcohol drinking on genetic and/or environmental variance of BMI using gene-environment interaction models. First, we conducted univariate structural equation model to estimate the genetic and environmental influences on BMI variance. Table 3 shows that the additive genetic/common environment/unique environment (ACE) model offered the best fit for BMI: dropping C from the model decreased the fit statistically significantly. Thus ACE model was used in the subsequent modeling. After adjusting for age and region, the estimate of heritability for BMI was 58 %.

**Table 3:** Genetic and environmental components of variance for BMI (*N* = 12242)^a^

	Proportion of variance (95 % CI)			Fit of model^b^		
	A	C	E	−2LL	AIC	*p*
ACE model	0.58 (0.52 0.64)	0.26 (0.20 0.31)	0.16 (0.16 0.17)	54846.73	30374.73	---
AE model	0.74 (0.73 0.75)	---	0.26 (0.25 0.27)	55617.62	31143.62	< 0.001

*Abbreviations: −2LL −*2 log likelihood, *A* additive genetic component, *C* common environment component, *E* unique environment component, *CI* confidence interval

^a^Adjusted for age and region in the model

^b^Model fitting comparison when shared environment component C is removed

Table 4 shows the moderating effects of current moderate to heavy cigarette smoking and current alcohol drinking on variance components of BMI. The variance explained by the genetic component did not significantly differ between moderate to heavy cigarette smokers and nonsmokers indicating no evidence of a gene-smoking interaction on BMI.

**Table 4:** Moderating effects of cigarette smoking and alcohol drinking on variance components of BMI^a^

	Additive genetic modification		Common environment modification		Unique environment modification	
	β_a_	95 % CI	β_c_	95 % CI	β_e_	95 % CI
Moderate to heavy cigarette smoking	−0.044	(−0.126 0.040)	0.127	(0.004 0.253)	0.014	(−0.005 0.033)
Current alcohol drinking	−0.137	(−0.215 -0.058)	0.187	(0.077 0.302)	−0.005	(−0.023 0.014)

β_a_: Moderating effect on additive genetic component; β_c_: Moderating effect on common environment component; β_e_: Moderating effect on unique environment component;

*Abbreviations: CI* confidence interval, *BMI* body mass index

^a^Adjusted for age and region in the model

Alcohol drinking significantly decreased genetic variance (β_a_ = −0.137; 95 % CI, −0.215 to −0.058) and increased common environmental variance (β_c_ = 0.187; 95 % CI, 0.077 to 0.302) of BMI suggesting that the heritability of BMI decreased in alcohol drinkers. These results indicated that in alcohol drinkers, environmental influences were predominant while genetic influences were suppressed.

## Discussion

This was the first study using twins to examine the associations of cigarette smoking and alcohol drinking with BMI in China. In the current study, after controlling for multiple socio-demographic and lifestyle factors, cigarette smoking and alcohol drinking were associated with BMI. Using within-pair analyses to exclude effects of shared factors, we found that moderate to heavy cigarette smoking was associated with decreased BMI while current alcohol drinking had no effect on BMI. Gene-environment interaction analyses showed no gene-smoking interaction on BMI, suggesting an immediate effect of cigarette smoking on BMI. Comparatively, we found that genetic variance of BMI decreased whereas common environmental influences predominated as a function of alcohol drinking.

A major finding in this study was that moderate to heavy smokers had lower BMI while former smokers had higher BMI than nonsmokers even when twins were considered as pairs. This finding is consistent with a previous twin study conducted in Vietnam-era twins [27]. The result that cigarette smoking as a continuous measure was also negatively associated with BMI is in accordance with another study conducted in Chinese population [28]. Compared with standard epidemiological studies, twin design provides a unique model to assess the quantified effects of environment factors on health outcomes with powerful control for genetic and shared environmental confounding factors. The inverse relationship between cigarette smoking and BMI may be explained by the biological function of nicotine. Nicotine during cigarette smoking acutely increases energy expenditure and reduces appetite [29] which could explain the lower body weight found in current smokers. Besides, a recent genetic meta-analysis indicated that the genetic variation in relation to the quantity of smoking was associated with a lower BMI in those who smoke, but not in those who have never smoked [30] also supported our finding. Given to the weight gain after smoking cession, our result additionally suggested that health practitioners should also consider routinely offering a weight management plan to reduce weight gain when advocating the smoking cessation campaigns.

Current alcohol drinking was associated with increased BMI in our analyses when treating twins as individuals, but the association did not remain significant in within twin-pair analyses of both MZ and DZ twin pairs indicating that shared familial factors may contribute to the association. In keeping with our findings, previous twin studies [27, 31] found daily alcohol consumption had no significant influence on weight or obesity. Studies have also reported that different types and quantity of alcohol consumption might have distinctive biologic effects. For example, a meta-analysis found that light-to-moderate wine intake protected against weight gain whereas consumption of spirits stimulated weight gain [11]. However, in this study, no information was collected in the questionnaire regarding various alcohol types and quantity of alcohol intake. As a result, types and quantity of alcohol consumption could not be distinguished. It is possible that their effects cancel each other out when combined together.

Another main result focused on whether moderate to heavy smoking and current alcohol drinking modified the genetic and environmental variance of BMI. We found no gene-smoking interaction on BMI. To date, no twin studies have examined the modification of smoking on BMI using similar models. However, genomics studies on the effects of smoking on the genetic risk of obesity supported our finding. A recent study indicated that there was no strong evidence that smoking status modified genetic effects of previously identified genetic risk factors for BMI in African Americans and European Americans [32]. Similar results have also been reported in British adults [33]. However, Edwards et al. [15] showed that gene-environment interactions were observed with cigarette smoking and a SNP in ADIPOR1, a BMI-related gene, in African Americans and indicated the effect of ADIPOR1 variation was stronger in nonsmokers and was greatly reduced in smokers. Therefore, more research is required to elucidate whether there is a BMI-related gene-smoking interaction in Chinese population.

By contrast, we found evidence that current alcohol drinking reduced genetic variance while increased common environmental variance of BMI. Common environment represents all environmental exposures that are not unique to an individual twin. Common examples include in utero exposures, birth history, diet, and childhood living conditions and location. In this study, the majority of the individuals are married and the average age of the population is 39 years. Although the twin pairs have not shared a common environment for many years, according to our result of gene-environment interaction, we hypothesized that in current drinkers, the environment for obesity-related gene expression is decreased, allowing behaviors learned earlier in life (such as meal timing and composition, lifestyle, and physical activity levels) to surface and drive body weight. Using a sample of female twins, Jerry et al. [16] found that the association between alcohol intake and abdominal adiposity was limited in twins genetically susceptible to obesity. This finding supported the idea of gene-alcohol interaction in influencing the risk of obesity. It is, however, unclear whether the result is applicable in males. Although the possible underlying mechanism has not been clarified completely, there is a possibility that the magnitude of the overall effect of obesity-related genes reduced due to the intake of alcohol. To our knowledge, evidence on gene-alcohol interactions in determining obesity is scarce and only one study reported that the minor allele of peroxisome proliferator-activated receptor-gamma coactivator 1, alpha (PPARGC1A) rs4619879 in combination with increasing alcohol consumption was associated with increased BMI among African Americans [15]. This result suggested a higher obesity-related gene expression in alcohol drinkers compared with nondrinkers, which was in the opposite direction of our finding. One of the reasons for this difference may be that this study focused on only a few of candidate genes while our result addressed the overall effects of genetic modification of BMI by alcohol drinking. Gene-alcohol interaction may be particularly beneficial in discovering potential candidate genes and contribute to find the missing heritability for BMI [34] as our result suggested heritability of BMI was lower in current alcohol drinkers when compared with nondrinkers. It is likely that significant associations will be masked in GWA studies including populations with differences in their alcohol exposure. Further studies with more careful assessment of alcohol intake using genetic predisposition score derived from obesity GWA data is required to robustly confirm our finding.

### Study limitations

The study has several limitations. Firstly, the data on BMI, cigarette smoking and alcohol drinking were based on self-reports. BMI based on self-reported height and weight may be inaccurate. In this study, however, the correlations between self-reported and measured weight and height values were high at about 0.90. Although studies reported that self-reported cigarette consumption was remarkably valid, misclassification may still remain. In addition, we had no detailed information on energy intake and other physical activity patterns like occupational physical activity, the possibility of residual confounding by these unmeasured covariates cannot be excluded. At last, we were not able to analyze the direction of causation due to cross-sectional design.

## Conclusions

In summary, using this population-based large male twin sample, we demonstrated that independent of genetic influence, cigarette smoking was negatively associated with BMI while smoking cessation was positively associated with BMI. Gene-environment interaction was found between alcohol drinking and BMI, with a down-regulation of genetic effects of BMI in current alcohol drinkers. Our results highlighted gene-environment interactions in elucidating the etiological factors of obesity. Further studies are needed to identify the potential BMI-related genes that interact with alcohol drinking. Furthermore, continued follow-up of this cohort would provide more insight into the relation and help to test the causality between cigarette smoking and BMI.

## Abbreviations

A: additive genetic component
ACE model: additive genetic/common environment/unique environment model
ADIPOR1: adiponectin receptor 1 gene
AIC: Akaike Information Criterion
BMI: body mass index
C: common environment component
CNTR: Chinese National Twin Registry
DZ: dizygotic
E: unique environment component
GWA study: genome-wide association study
MZ: monozygotic
PPARGC1A: peroxisome proliferator-activated receptor-gamma coactivator 1, alpha
